# Smart Electronic Eyedrop Bottle for Unobtrusive Monitoring of Glaucoma Medication Adherence

**DOI:** 10.3390/s20092570

**Published:** 2020-04-30

**Authors:** Marcelo Aguilar-Rivera, Dieanira T. Erudaitius, Vincent M. Wu, Justin C. Tantiongloc, Dae Y. Kang, Todd P. Coleman, Sally L. Baxter, Robert N. Weinreb

**Affiliations:** 1 Department of Bioengineering, University of California San Diego, La Jolla, San Diego, CA 92093, USA; mia003@eng.ucsd.edu (M.A.-R.); derudait@gmail.com (D.T.E.); v5wu@eng.ucsd.edu (V.M.W.); daeykj@gmail.com (D.Y.K.); tpcoleman@ucsd.edu (T.P.C.); 2 Department of Computer Science and Engineering, University of California San Diego, La Jolla, San Diego, CA 92093, USA; jctanti@gmail.com; 3 Hamilton Glaucoma Center, Viterbi Family Department of Ophthalmology and Shiley Eye Institute, University of California San Diego, La Jolla, San Diego, CA 92093, USA; s1baxter@health.ucsd.edu; 4 Health Department of Biomedical Informatics, University of California San Diego, La Jolla, San Diego, CA 92093, USA

**Keywords:** glaucoma, adherence, monitoring, sensors, alerts, clinical decision support, internet of things

## Abstract

Glaucoma, the leading cause of irreversible blindness, affects >70 million people worldwide. Lowering intraocular pressure via topical administration of eye drops is the most common first-line therapy for glaucoma. This treatment paradigm has notoriously high non-adherence rates: ranging from 30% to 80%. The advent of smart phone enabled technologies creates promise for improving eyedrop adherence. However, previous eyedrop electronic monitoring solutions had awkward medication bottle adjuncts and crude software for monitoring the administration of a drop that adversely affected their ability to foster sustainable improvements in adherence. The current work begins to address this unmet need for wireless technology by creating a “smart drop” bottle. This medication bottle is instrumented with sensing electronics that enable detection of each eyedrop administered while maintaining the shape and size of the bottle. This is achieved by a thin electronic force sensor wrapped around the bottle and underneath the label, interfaced with a thin electronic circuit underneath the bottle that allows for detection and wireless transmission to a smart-phone application. We demonstrate 100% success rate of wireless communication over 75 feet with <1% false positive and false negative rates of single drop deliveries, thus providing a viable solution for eyedrop monitoring for glaucoma patients.

## 1. Introduction

Reduced adherence with prescribed systemic and topical medications (such as eyedrops) for treating chronic illness has long been identified as a key obstacle to delivering successful treatment. As the World Health Organization (WHO) has declared, “Increasing the effectiveness of adherence interventions may have a far greater impact on the health of the population than any improvement in specific medical treatments” [1].

Reduced adherence is a problematic issue for the management of glaucoma, a chronic neurodegenerative eye disease that is the leading cause of irreversible blindness globally, projected to affect now more than 80 million people worldwide [2,3]. Lowering intraocular pressure (IOP) is the only proven method of delaying both the development and progression of glaucoma [4]. The most widely employed first line therapy is achieved by topical administration of a series of eye drops to reduce intraocular pressure (IOP) [5,6,7]. Patients need to administer eye drops daily, often multiple times, and more than one half of patients administer more than one type of eye drop. With an often bewildering regimen of eye drop use, it is not surprising that adherence with eye drops ranges from 30% to 80% [8,9,10]. These levels fail to meet the 80% threshold recognized as an acceptable standard of adherence for many systemic medications [11]. Moreover, patients tend to overestimate their own adherence compared to device-measured or pharmacy refill data [12]. Glaucoma medication non-adherence can hasten disease progression and lead to worsening visual impairment and eventual blindness [13,14,15,16,17]. Prior studies have demonstrated that patient education alone is not sufficient for improving adherence [18].

Early evidence indicates a potential role for the use of alerts or reminders at drop-taking times, bolstered by the widespread use of smart-phone enabled technologies [19,20,21,22,23]. However, previous electronic monitoring solutions for eye drops have design drawbacks. For example, a dosing aid [24] had awkward medication bottle adjuncts and crude software for monitoring the administration of a drop. More recently, customized eyedrop bottles have been developed that contain sensors and electronics for wireless signaling of adherence patterns to smart phones for reminders [25]. However, this system utilizes custom bottles of larger size than typical eyedrop bottles, and therefore makes it inconvenient for the patients to carry. There is an unmet need for a seamlessly integrated technology that can register a successful drop delivery to the eye and communicate this information to both patients and providers [26]. Ideally, such a measurement apparatus should not change the shape or size of the eyedrop bottle. 

We have developed an electronic adherence monitoring system capable of addressing these unmet needs that offers several innovations over existing technologies. Here, we report the performance of this system under a wide range of testing conditions to demonstrate its accuracy, reliability, and feasibility for clinical deployment. Specifically, we demonstrate a 100% success rate of wireless communication over 75 feet with false positive and false negative rates of single drop deliveries below 1%.

## 2. Materials and Methods

### 2.1. Fully Integrated Electronic Eyedrop System

**Figure 1:**
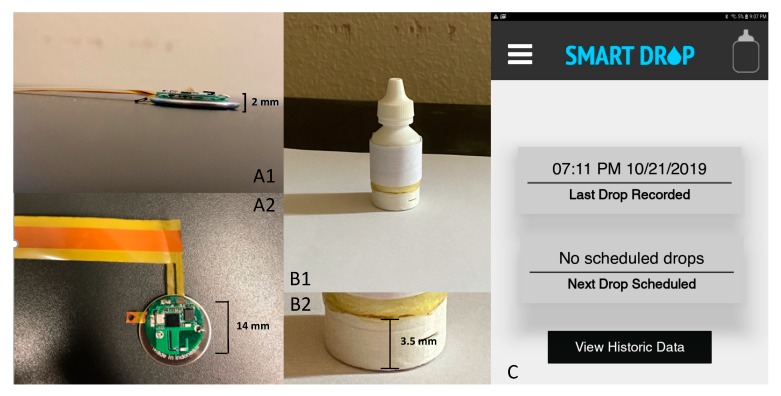
(**A1**) Lateral view of electronics underlying the smart drop system, comprising a thin conductive pressure-sensitive electronic sensor, for bottle squeezing detection and an electronic circuit (<2 mm thick) for signal processing and wireless transmission. (**A2**) Top view of electronic sensor and electronics placed beneath the bottle. The circuit diameter and battery are approximately 14 mm in diameter. (**B1**) An eyedrop bottle containing the flexible sensor underneath the label, and electronics at the base of the bottle. (**B2**) Plastic case (height 3.5 mm) underneath an eyedrop bottle that covers the battery and the circular electronic circuit. (**C**) Smart phone application that communicates via Bluetooth Low Energy (BLE) with the instrumented eyedrop bottle to track eyedrop adherence. The application can update patient adherence information to a database (Google) where individuals (e.g., physicians, care providers, family members) can track how the patient adheres. The database also allows for real-time updating of reminder specifications on the smart phone app, so that programed reminders for patients can be modified remotely.

The prototype of smart eyedrop system consists of a standard eyedrop bottle (height: 5.7 cm, diameter: 2.4 cm) outfitted with flexible electronics to detect when the bottle is squeezed. This system builds upon recently demonstrated multi-functional uses of thin and flexible electronics for medical monitoring [27,28], which in this case address the key unmet need of measuring glaucoma eyedrop adherence in a manner that does not alter the shape or size of the bottle. As shown in Figure 1B, a flexible passive sensor for bottle squeeze detection is placed under the adhesive label of the eyedrop bottle. The sensor contains two electrically conductive sheets (copper, 15 um thick) separated by a adhesive tape with an insulator (cellulose acetate, 38 um thick) that is doubly coated with an adhesive (acrylic, 22 um thick). The adhesive tape is perforated with 4 mm holes allowing for electrical connection between the two electrically conductive sheets upon force.

The flexible sensor is interfaced with a circular printed circuit board of 0.5 mm thickness placed underneath the bottle. A programmable system on a chip (PSOC^®^ 4, Cypress Semiconductor, San Jose, CA, USA) contains a microprocessor and Bluetooth low energy module to process sensor data and allow for communication wirelessly to the smart phone application. The two electrically conductive sheets are interfaced to pins of the PSOC, one of them to ground, so that upon a bottle squeeze, a digital signal transitions from one an open circuit to short circuit. Upon a bottle squeeze, the microprocessor reads from a gyroscope (Bosch Sensortec, Inc., Reutlingen, Germany) with I2C communication to determine whether or not the bottle is tilted upside down. Only in that context is a bottle squeeze indicative of an eyedrop registered, thus decreasing the rate of false positives as discussed below. A coin cell (CR2016), also beneath the eyedrop bottle, powers the system; power consumption and its relation to usability within the context of eyedrop adherence are discussed below. Our system is easily adjustable and customizable to different bottle sizes and shapes, as well as battery types. Further, we can adjust the behavior of the passive flexible sensor without further modifying the electronics by varying the size of, and distance between, the windows in the plastic layer that separates the two conductive sheets that form the sensor.

### 2.2. Mobile Application for Monitoring Drops and Adjusting Reminders

Upon delivery of a drop, the electronic eyedrop bottle transmits a signal via BLE to a mobile device (e.g., a smart phone or tablet). Given that the application (Figure 1C) is configured so that a patient can specify reminder settings on their smart device, upon delivery of a drop, the reminder for the time window of interest is de-activated upon delivery of the drop. The information from the mobile device is also connected to a cloud-hosted real-time database (Google Firebase Realtime Database) that is also synchronized with any other connected application. This allows for allows for doctors, caregivers, or families to obtain up-to-date information about a patient’s adherence when using an application connected to the database. In addition, the mobile application is configured so that reminders are adjustable through the real-time database and can be adjusted from other applications with privileges to interface with the database, thus allowing for a care provider or family member to remotely alter suggested reminders based upon a patient’s recent adherence progress.

### 2.3. Smart Eyedrop Bottle Behavior

**Figure 2:**
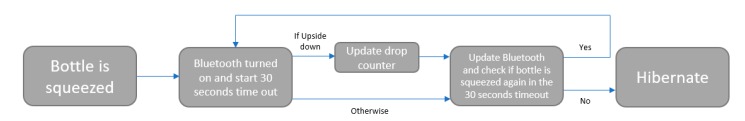
Scheme depicting normal behavior of the eyedrop bottle system. When upside-down bottle is squeezed, the system broadcast such the time stamp information of when the bottle was squeezed.

The smart eyedrop bottle is designed to reduce false positives and save energy for identifying attempts at drop administration by activating the microprocessor and wireless system only when the bottle is tilted downward and the bottle is squeezed, thus avoiding situations when the bottle is pressed upon in a purse or bag while someone is walking. The software operation is described in Figure 2, where it is possible to see that when the bottle is squeezed while tilted downward, the BLE system wakes up and sends the information about the squeezing to the mobile application. If the bottle is not pressed during the 30-s timeout, the bottle system hibernates in order to save energy. It is possible to customize the ‘timeout’ to extend the battery life of the system. 

### 2.4. Smart Eyedrop Bottle Performance Testing

A range of tests were performed on the eyedrop bottle sensor prototypes in a laboratory environment. These tests were aimed at validating the accuracy of the sensor and additionally evaluating readiness for clinical deployment. 

Battery Consumption Test: A key objective of the design of the sensor prototype was to ensure adequate battery life for clinical testing. While some glaucoma medications are dosed once daily, several classes of glaucoma medications are dosed twice or three times daily. In addition, patients with glaucoma are often undergoing simultaneous treatment for both eyes. Therefore, to simulate maximal usage, we decided to test the prototype sensor with six delivery events daily. We recorded the battery life of CR2016 among six different bottles to ascertain the average battery life of the prototype. Battery life was defined as the number of days between the first day of delivery events and the last day when a delivery event was successfully registered and transmitted. The final voltage of each bottle’s battery was also recorded. 

Distance Tests: To evaluate the maximum distance at which wireless transmission of a medication delivery event between the sensor on the bottle and the application could be achieved, testing was performed with varying distances between the sensor prototype and the tablet where the user interface application was installed. This simulates real-world conditions where patients may not always be administering their eye drops immediately adjacent to a smart phone or tablet containing the application. Two variations of distance testing were performed: (1) a straight distance test and (2) a test through a door/wall. The straight distance test was used to illustrate the maximum distance for successful communication between the bottles and the application. To perform this test, a tablet with the application installed was placed on a table. Then, each bottle was squeezed every 5 ft as it was moved away from the tablet until it disconnected from the application. The distance at which the bottle became disconnected from the application was measured and recorded for two bottles with five repetitions each. The second variation of the test, the “through the door/wall test”, was performed similarly but with a door and wall positioned between the tablet application and the bottle sensor. This further simulated the home environment, where patients may be using glaucoma medications in a separate room than where their tablet or smart phone may be located. The second variation of the distance test was performed on the same bottles as the first variation, in order to directly evaluate the effect of intervening physical structures on the connectivity of the sensor. 

False Positive and False Negative Tests: To ensure that the sensor would record only true medication delivery events rather than arbitrary movements, we performed the following sequence of tasks: we placed the bottle in a packed bag, turned the bag upside down and manually shook it for 5 s, walked around and then dropped the bag on the ground. The tablet application was then analyzed to evaluate for any registration of medication delivery events during this sequence when the bottle was not intentionally squeezed (i.e., to evaluate for any false positives). These false positive tests were performed on two bottles for 10 times each. 

Two iterations of false negative testing were done to demonstrate that the application would not fail to register medication delivery events. First, each bottle used in the movement sequence above was intentionally squeezed, and the application was analyzed to evaluate for successful registration. This also demonstrated whether any of the bottles were damaged during the false positive test. In the course of working with the bottles, we also incidentally noticed that if the bottle is squeezed multiple times in quick succession, the application may not register all the squeezes. Therefore, we developed another iteration of a false negative test by squeezing the bottles two times separated by 0.5, 1, 2, and 3 s to observe how the system would react. These false negative tests were performed on two bottles for five repetitions each.

Temperature Test: Testing of connectivity between the sensor and the tablet application was also performed in various extremes of temperature. The objective was to evaluate whether the sensor could still function if placed in low temperature settings such as the refrigerator (which is a common source of medication storage for glaucoma patients, who sometimes use the cold sensation to help gauge whether an eyedrop has reached their eye successfully). Two bottles were tested for five repetitions each for the low temperature conditions. Bottles were placed in a 3 °C refrigerator for 2, 4, 6, 8, and 14 h, with the 14-h setting simulating overnight storage conditions. After each period of cold exposure, we removed the bottle from the refrigerator and immediately squeezed once to evaluate the connectivity to the tablet.

For each test, descriptive statistics were generated using Microsoft Excel (Redmond, WA, USA). Data from the tests were analyzed and visualized using R [29] and Python.

## 3. Results

### 3.1. Battery Consumption Test 

**Figure 3:**
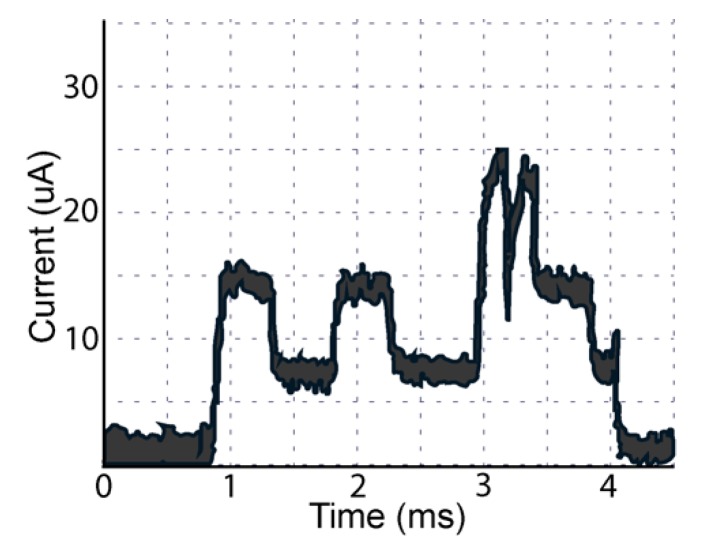
Each bottle squeeze consumes ~12 μA for 3 ms. Intact bottles can achieve >1000 pushes and BLE transmissions while battery voltage is over 1.8 V, the minimum input voltage for the Cypress programmable system on a chip (PSOC).

The BLE radio in our electronic eyedrop system consumed most of the energy during eyedrop adherence monitoring. Each BLE transmission occurred at a frequency of 33 Hz for a period of 30 s after the bottle was squeezed, when the PSOC was awake. Figure 3 illustrates the current battery consumption result from one single bottle squeeze. It was composed of three consecutive spikes and repeated with a frequency of 33 Hz for 30 s.

Firmware optimization for which microprocessor activation did not ensue until the gyroscope and squeeze sensor cross thresholds strategically allowed us to optimize the battery life of a CR2016 to withstand over 1000 bottles squeezes, thus vastly exceeding the three weeks of six times daily. 

**Figure 4:**
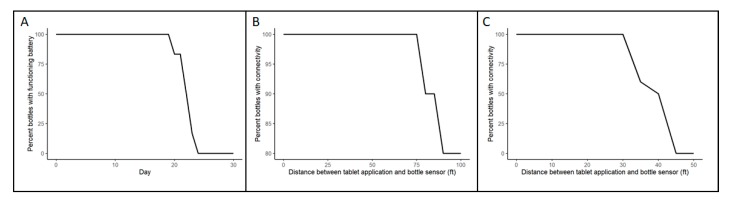
(**A**) Each bottle was squeezed six times per day. The average number of days a bottle operates with a functional battery 21.3 days. (**B**) A straight distance test for Bluetooth connectivity between the bottle and a tablet; bottle connection rates for 75 ft and below are 100%. (**C**) A test of connectivity with door or wall between the bottle and tablet application; distance of successful medication delivery event registration was 36 ± 4.7 ft.

In a simulation of maximal clinical usage of glaucoma medications dosed three times daily for both eyes (for a total of six times daily), the tested bottles (n = 6) had a mean (standard deviation, SD) battery life of 21.3 (1.3) days, ranging from 19 to 23 days (Figure 4A). The PSOC preserved radio functionality while battery voltage was over 1.8 V.

### 3.2. Distance Tests

Among tested bottles that were progressively moved farther from the tablet application, successful medication delivery events were recorded up to a mean (SD) distance of 96 (8.3) feet (range: 75–100 feet). All (100%) bottles were successfully connected at 75 feet, and 80% of bottles were successfully connected even at 100 feet (Figure 4B). 

When a door or wall was interposed between the tablet application and the bottles, the mean (SD) distance of successful medication delivery event registration was 36 (4.7) feet (range 30–40 feet). In this circumstance, 100% of the bottles were successfully connected within 30 feet, and then connectivity rates declined as bottles were moved to progressively farther distances (Figure 4C).

### 3.3. False Positive and False Negative Tests

Despite rigorous movement involved in the sequence of testing done for evaluation for false positives (see Methods Section for details), the tablet application did not register any medication delivery events in the absence of intentional squeezing to deliver a medication, representing a false positive rate of 0% after 20 runs. In each run of the test, the connectivity of the sensor was verified, ensuring that the false positive rate was truly 0% and not just a result of absent or faulty connectivity between the sensor and the tablet application.

**Figure 5:**
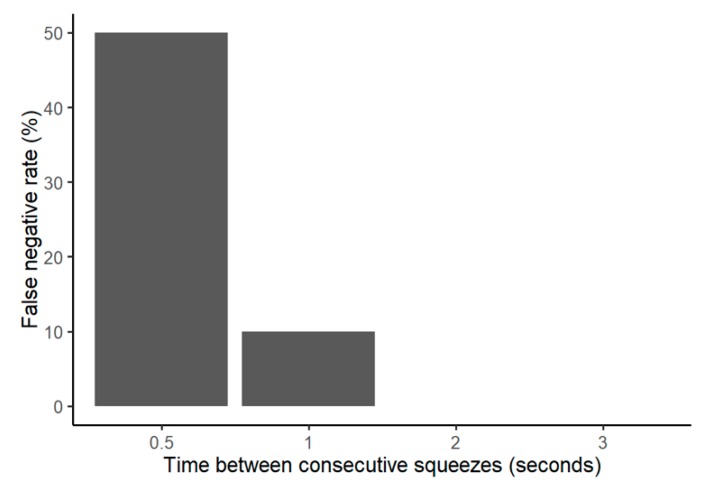
False negative test with two consecutive squeezes. For 2 and 3 s between squeezes, the false negative rate is at 0%.

There were no false negatives in the tablet application when the bottles were squeezed only once. However, if the bottles were squeezed two times in quick succession, the tablet application did not always register the second squeeze, representing false negatives. The false negative rate was 50% if the two squeezes were separated by 0.5 s and decreased to 10% once the two squeezes were separated by 1 s (Figure 5). The false negative rate decreased to 0% once the time between squeezes was 2 s or longer. 

### 3.4. Temperature Tests

The sensor bottles were tested at low temperatures (3 °C). Every bottle was still fully functioning and able to successfully register medication delivery events in the tablet application even after 14 consecutive hours. 

## 4. Discussion and Conclusions

We have developed a fully functioning eyedrop bottle prototype that can successfully transmit data wirelessly to a smart device and document when a drop is properly administered. This innovative apparatus can monitor glaucoma medication adherence and is been patent pending.

Our data demonstrate the feasibility of this prototype for real-world deployment. First, the battery life averaged approximately 21 days, allowing three weeks of adherence data collection, which in most cases would capture a representative slice of patient behavior. Rarely in ambulatory clinical practice are health data obtained daily (or multiple times daily) for consecutive weeks. Supplying three weeks’ worth of data represents an excellent starting point. In the future, we will work toward extending battery life or developing recharging capabilities such that the sensor can continue recording adherence data until the patient runs out of medication in the bottle. There are many ways to achieve it; one straight-forward way to do so is to use a more efficient Bluetooth chip, which consumes less current and operates at lower voltages, allowing for more efficient BLE transmission.

Another consideration is athat the battery life testing was performed under the scenario of maximum dosage delivery (six times daily). Several classes of glaucoma medications are dosed just a few times daily and would therefore entail less frequent squeezes. A reduction on the dosage based on the type of medication and the hardware upgrade described above would extend battery life. In clinical practice, the battery life of our eyedrop bottle system would stisfy the adherence monitoring period of one month for all patients who use a given medication no more than four times daily. 

Connectivity between the sensor on the bottle and the tablet application storing the adherence data was maintained at distances over 100 feet in an open space, but the connectivity decreased to an average of 36 feet if there was an intervening door or wall between the bottle and the tablet. It is possible moving forward to use more efficient BLE protocols (e.g., Bluetooth 5.0) that allow for longer transmission distance with less power.

In initial deployment, it may be sensible to advise patients to administer their eyedrops within the same physical room as the device (smartphone or tablet) running the application, as ~30 feet would encompass the dimensions of the majority of indoor rooms in private homes. However, future iterations may allow for longer-range data transmission, such as using another Bluetooth system with optimized antennas for which patients could use their drops in any room and would not need to be physically near their device in order for adherence data to register. 

Several other features support this prototype’s readiness for the clinic. The false positive rate of the sensor was 0%, thus mitigating any concerns that dosages would be improperly recorded from eyedrop bottles being carried in patients’ purses or backpacks. The system only registered a dose as given if the bottle was in the correct orientation and squeezed with the appropriate force. Similarly, the false negative rate was 0% for single squeezes. Although there were some false negatives for squeezes in quick succession (separated by 1 s or less), this would not represent a major issue, since in the context of typical patient use, multiple successive squeezes often constitute a single “dose” anyway. Furthermore, the sensor demonstrated successful connectivity in low-temperature settings, illustrating that adherence data would be successfully collected even if the patients store their eyedrops in the refrigerator. In short, these results suggest that the sensor will be able to perform not only in the laboratory, but also in real-world environments. 

Furthermore, the costs of deploying this system in clinical environments are not prohibitive. Whereas the cost of manufacturing our prototype is currently approximately $100, we anticipate that widespread clinical adoption in high volumes along with ongoing technological trends will allow for manufacturing costs to not exceed $1 per unit. 

This technology offers several key advantages over current practice. First, most clinicians monitor adherence by interviewing patients and acquiring self-reported data. Unfortunately, several studies have shown that patients’ self-reported adherence is often overestimated. Another method of monitoring adherence is examining claims data or medication dispensing data. This can be difficult if the patient has multiple forms of insurance and/or uses multiple pharmacies, both of which are not uncommon scenarios. In addition, claims and dispensing data do not have the level of dose-to-dose granularity. Finally, previously reported electronic dosing trackers for eye drops have all required separate hardware and often bulky designs that limit widespread adoption. Using an unobtrusive device that is integrated with the eyedrop bottle itself to gather data on individual dose adherence provides a source of objective and granular data to help guide glaucoma management. Future studies to better understand how patients will use and interact with this technology and how clinicians will integrate these new data streams into their workflows will be critical. 

Our next steps are to continue minimizing the system to have a fully integrated system that fits unobtrusively beneath the bottle label, as well as extending the battery life of the system. In addition, we are conducting ongoing work to validate its performance on different surfaces to ensure consistency of readings across a wide range of eyedrop bottles. In doing so, we will have a fully deployable prototype for future pilot studies to evaluate improvement of glaucoma adherence. We have already developed clinical protocols for testing these sensors among patients at the glaucoma clinics of the University of California San Diego (UCSD) Shiley Eye Institute that have been approved by the UCSD Institutional Review Board. Adherence data collected by the sensors will be compared with patient self-report. We will perform statistical modeling to understand how sensor-acquired adherence data are associated with measures of patients’ glaucoma progression. We will also conduct interviews with patients to assess the usability of the device. Ultimately, we plan to develop an adherence dashboard for clinicians and a patient-facing mobile application to enable real-time dosing reminders and other forms of patient engagement in order to improve adherence.

Altogether, we provide a unique and promising tool for monitoring and fostering glaucoma patient adherence, with the goal of enhancing provider-patient communication and patient engagement to improve outcomes, ultimately reducing the burden of irreversible blindness of advanced glaucoma.
